# Resting-state functional connectivity between the dorsal anterior cingulate cortex and thalamus is associated with risky decision-making in nicotine addicts

**DOI:** 10.1038/srep21778

**Published:** 2016-02-16

**Authors:** Zhengde Wei, Nannan Yang, Ying Liu, Lizhuang Yang, Ying Wang, Long Han, Rujing Zha, Ruiqi Huang, Peng Zhang, Yifeng Zhou, Xiaochu Zhang

**Affiliations:** 1 Key Laboratory of Brain Function and Disease, Chinese Academy of Sciences, School of Life Sciences, University of Science & Technology of China, Hefei, Anhui 230027, China; 2 Provincial Hospital Affiliated to Anhui Medical University, Hefei, Anhui 230001, China; 3 State Key Laboratory of Brain and Cognitive Science, Institute of Biophysics, Chinese Academy of Sciences, Beijing, 100101, China; 4 School of Humanities & Social Science, University of Science & Technology of China, Hefei, Anhui 230026, China; 5 Center of Medical Physics and Technology, Hefei Institutes of Physical Science, CAS, Hefei, Anhui 230031, China; 6 Centers for Biomedical Engineering, University of Science & Technology of China, Hefei, Anhui 230027, China

## Abstract

Nicotine addiction is associated with risky behaviors and abnormalities in local brain areas related to risky decision-making such as the dorsal anterior cingulate cortex (dACC), anterior insula (AI), and thalamus. Although these brain abnormalities are anatomically separated, they may in fact belong to one neural network. However, it is unclear whether circuit-level abnormalities lead to risky decision-making in smokers. In the current study, we used task-based functional magnetic resonance imaging (fMRI) and examined resting-state functional connectivity (RSFC) to study how connectivity between the dACC, insula, and thalamus influence risky decision-making in nicotine addicts. We found that an increase in risky decision-making was associated with stronger nicotine dependence and stronger RSFC of the dACC-rAI (right AI), the dACC-thalamus, the dACC-lAI (left AI), and the rAI-lAI, but that risky decision-making was not associated with risk level-related activation. Furthermore, the severity of nicotine dependence positively correlated with RSFC of the dACC-thalamus but was not associated with risk level-related activation. Importantly, the dACC-thalamus coupling fully mediated the effect of nicotine-dependent severity on risky decision-making. These results suggest that circuit-level connectivity may be a critical neural link between risky decision-making and severity of nicotine dependence in smokers.

Nicotine addiction is associated with risky behaviors^1^,^2^ and abnormalities in brain areas related to decision making^3^,^4^ such as the dorsal anterior cingulate cortex (dACC)^5^,^6^, insula^7^,^8^, thalamus^9^,^10^ and striatum^11^,^12^.

The dACC is primarily responsible for evaluation in decision making^13^. Abnormal activity in the dACC has been linked to cravings for cigarettes^9^. The anterior insula (AI) plays a key role in mediating the relationship between nicotine dependence and risky decision making [e.g., Risky-Gains task^14^]. Abnormal activity in the thalamus has also been associated with cigarette cravings^15^. Although brain abnormalities associated with risky decision making and nicotine addiction are found in anatomically separated regions (e.g., the dACC, AI and thalamus), these regions may in fact be parts of one neural network. Changes in this neural network may reveal important information about global deficits in the organization of the brain^16^. So far, it is unclear whether circuit-level abnormalities lead to behavioral deficit in risky decision making in population of nicotine addicts.

The dACC, AI and thalamus are critical nodes of the salience network, a well-defined functional brain network^17^ that mainly serves to identify relevant internal and external stimuli to guide behavior^18^. Risky stimuli (e.g., a risky choice), which are associated with strong subjective excitement and strong interoceptive change^19^, may be highly salient. Previous studies found stronger connectivity within the salience network in nicotine addiction^20^,^21^. In the present study, we hypothesized that in population of nicotine addicts, greater connectivity between the dACC, AI, and thalamus may enhance the salience processing of risky stimuli, resulting in an increase in risky decision making.

To study how functional connectivity influence risky decision making in population of nicotine addicts, we used task-based functional magnetic resonance imaging (fMRI) in conjunction with the Balloon Analogue Risk Task (BART^22^; Fig. 1) and resting-state functional connectivity (RSFC), which can acquire temporal correlations between spontaneous regional activities in the absence of externally controlled tasks or stimuli^23^. We first tested the relationship between the severity of nicotine dependence and risky decision making using BART. Then, we used correlation and causal mediation analyses, statistical methods that can inform understanding of how brain regions interact to result in behavior, thus, whether the RSFC was the link between risky decision making and the severity of nicotine dependence.

## Results

### Task performance

**Figure 2:**
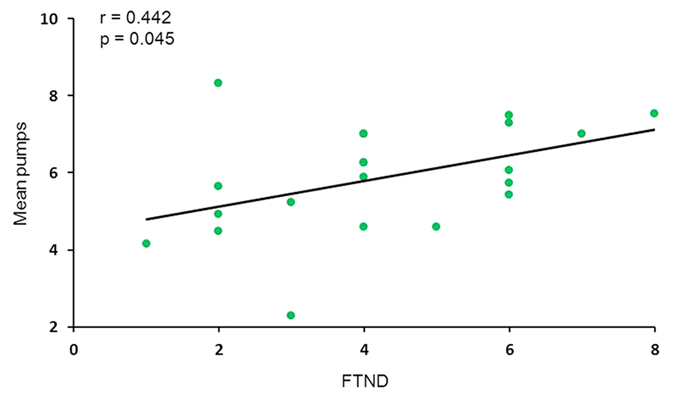
The mean pumps positively correlated with nicotine dependence severity.

The BART used in this study is a sequential decision-making task, widely used as a tool for assessing risky behavior^22^. It provides participants a chance to pump virtual balloons to win potential monetary reward. However, each pump simultaneously increases the risk to lose accumulated reward for that balloon^22^. The mean pumps (the mean number of pumps across trials for balloons that did not explode, which is a typical behavior index of risky decision making in the BART) were 5.7 ± 1.4 and the FTND (Fagerström Test of Nicotine Dependence, which is used to measure the severity of nicotine dependence) score was 4.2 ± 1.9. The mean pumps were positively correlated with the FTND scores across participants (r = 0.442, p = 0.045; Fig. 2).

### Relationship of task-based activation to task performance and severity of nicotine dependence

During pumping, significant risk level-related activations (p < 0.005, family-wise error corrected) were found in the bilateral dorsal anterior cingulate cortex (dACC), right anterior insula (rAI), left anterior insula (lAI), bilateral thalamus, right precentral gyrus, right inferior parietal lobe (rIPL) and left inferior parietal lobe (lIPL) (Table 1). None of the activations of these regions significantly correlated with the mean pumps or FTND scores (Table 1).

### RSFC and relationship to task performance and severity of nicotine dependence

**Figure 3:**
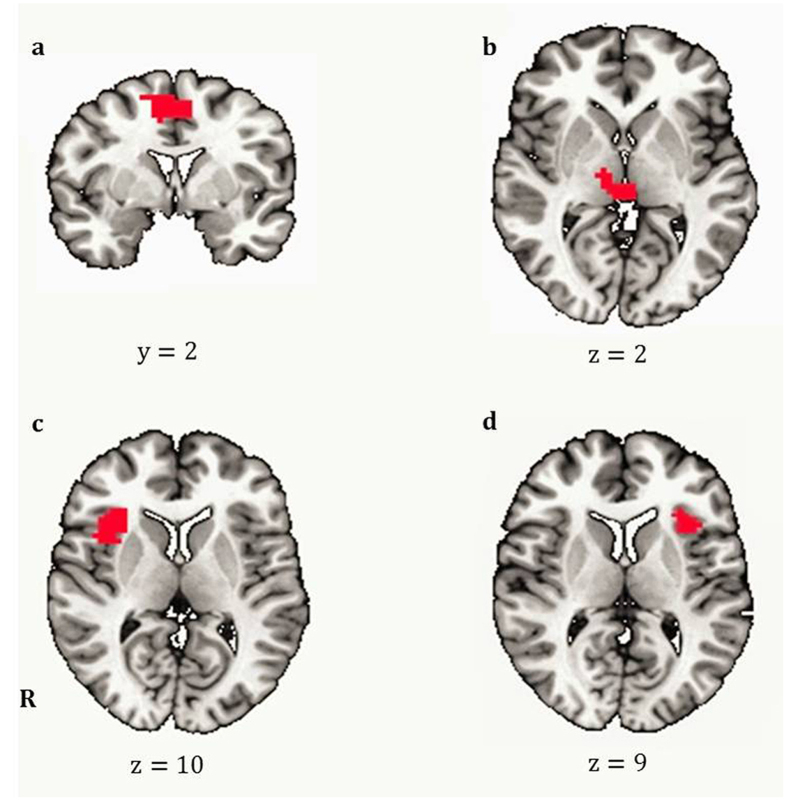
Significant risk level-related activation during risky decision making (regions of interest for RSFC). Panel (**a**). During risky decision making, risk level-related activation was found in the bilateral dorsal anterior cingulate cortex (dACC). Panel (**b**). Bilateral thalamus. Panel (**c**). Right anterior insula (rAI). Panel (**d**). Left anterior insula (lAI).

**Figure 4:**
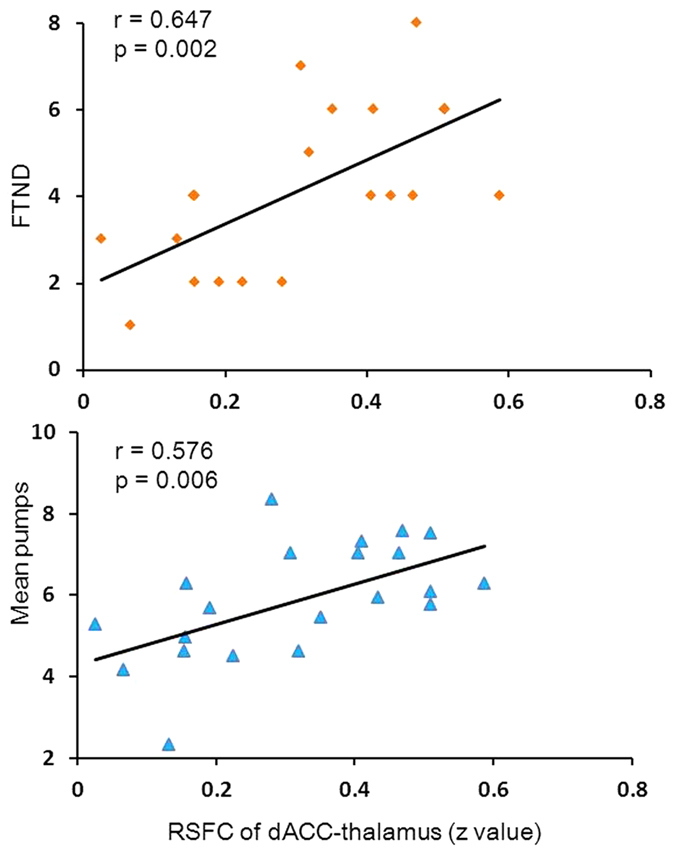
RSFC and its relationship to the FTND score and the mean pumps. The RSFC of the dACC-thalamus positively correlated with the FTND score and the mean pumps.

The RSFCs between the bilateral dACC, r-AI, l-AI and bilateral thalamus (Fig. 3) were calculated and the relationship to the mean pumps and FTND score was tested. Mean pumps positively correlated with the RSFC of dACC-rAI (r = 0.525, p = 0.015), dACC-thalamus (r = 0.576, p = 0.006), dACC-lAI (r = 0.564, p = 0.008), and rAI-lAI (r = 0.465, p = 0.034), but did not correlate with the other RSFC. The FTND score positively correlated with the RSFC of the dACC-rAI (r = 0.473, p = 0.031) and dACC-thalamus (r = 0.647, p = 0.002), but did not correlate with the other RSFC. We performed a FDR-correction for multiple comparisons (FDR < 0.05). Correlation between the mean pumps and the RSFC of dACC-thalamus (r = 0.576, p = 0.036, corrected), dACC-lAI (r = 0.564, p = 0.024, corrected), dACC-rAI (r = 0.525, p = 0.030, corrected), and rAI-lAI (r = 0.465, p = 0.051, corrected), and correlation between the FTND score and the RSFC of dACC-thalamus (r = 0.647, p = 0.012, corrected) survived the correction. However, correlation between the FTND score and the RSFC of dACC-rAI (r = 0.473, p = 0.093, corrected) did not survive. Only the RSFC of the dACC-thalamus correlated with both the mean pumps and the FTND score (Fig. 4).

Other RSFC between regions related to risk level except couplings between bilateral dACC, bilateral AI and bilateral thalamus were calculated and then relationship to mean pumps and FTND score was tested (see details in supplementary materials). We did not find any coupling that significantly correlated with both mean pumps and FTND score.

### RSFC mediation effect

**Figure 5:**
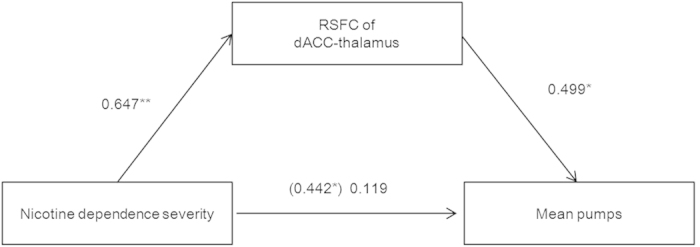
RSFC mediation effect. The RSFC of the dACC-thalamus fully mediated the effect of severity of nicotine dependence on the mean pumps. Values presented are the standardized regression coefficients. The value in parentheses represents the coefficient for the direct (i.e., unmediated) path. *p < 0.05, **p < 0.01, one-tailed.

Mediation analysis is a statistical method widely used in psychology research to examine correlated pathways among relational variables and tests whether association between an independent and dependent variable could be explained by a third variable^24^. To examine whether the RSFC of the dACC-thalamus pathway might mediate the effect of the FTND score on mean pumps, we performed a mediation analysis^24^,^25^ (see details in methods section). The mediation effect was considered to occur if the relationship between the FTND score (independent variable) and mean pump (dependent variable) could be partially or totally accounted for by the RSFC of the dACC-thalamus (hypothesized mediator). In the first step of the mediation analysis, the hypothesized mediator of the RSFC of dACC-thalamus was regressed on the FTND scores. Higher FTND score predicted a stronger RSFC of dACC-thalamus [β = 0.647, SE = 0.015, t(19) = 3.694, p = 0.001]. Secondly, the mean pumps were regressed on the FTND score, and the higher FTND score significantly predicted more mean pumps [β = 0.442, SE = 0.275, t(19) = 2.145, p = 0.023]. Thirdly, the mean pumps were regressed on the FTND score together with the hypothesized mediator. Stronger RSFC of dACC-thalamus significantly predicted more mean pumps [β = 0.499, SE = 2.151, t(18) = 1.990, p = 0.031]. The relationship between the FTND score and mean pumps decreased in strength with the presence of the mediator (RSFC of dACC-thalamus) and was not significant [β = 0.119, SE = 0.188, t(18) = 0.473, p = 0.321]. The mediation analysis indicated that the RSFC of dACC-thalamus fully mediated the effect of the severity of nicotine dependence on the mean pumps (Fig. 5). A partial correlation analysis was employed to support the mediation effect. The FTND score was not correlated with the mean pumps while controlling for the influence of RSFC in the dACC-thalamus pathway (r (18) = 0.111, p = 0.642) (see the detail in supplementary materials).

## Discussion

In the present study, we found that the FTND was positively associated with mean pumps. More importantly, greater RSFC among the dACC, AI, and thalamus was associated with higher mean pump values and more severely nicotine dependence in smokers.

Our results that showed association between greater RSFC of dACC-rAI, dACC-thalamus, dACC-lAI and rAI-lAI and increased risky decision making (i.e., the mean pumps) suggested that circuit-level engagement of the salience network may be required in risky decision making. One study has shown that the activity in a large network of brain regions tasked with processing risk during BART could predict choices in future risky decisions^26^. The dACC, AI, and thalamus have been suggested as important nodes of the salience network that respond to the degree of subjective salience, whether emotional, cognitive, or homeostatic^27^,^28^. Thus, it is possible that facilitated communication within the salience network is represented to enhance the processing of salient stimuli^29^.

Risk level-related activations of brain areas were not associated with mean pumps in the present study, which was consistent with previous studies using BART^2^,^4^,^30^,^31^,^32^,^33^. It is possible that because decision making employs a complex set of cognitive functions, circuit-level engagement possesses more explanatory power than the function of a single region. Furthermore, recent evidence suggests that decision making is mediated by a brain network centered on the dACC and that elements of the network are interconnected^13^. Functional networks, rather than single brain nodes, have amassed the ability to support complex functions such as decision making^34^.

Studies of resting-state^35^ and task-based^36^ functional connectivity in population of nicotine addicts have shown that connectivity with the dACC is modulated as a function of severity of nicotine dependence. However, whether the link between risky decision making and severity of nicotine dependence can be explained by changes in functional connectivity in population of nicotine addicts has not been directly tested. The present study found that the dACC-thalamus coupling fully mediated the relationship between severity of nicotine dependence and mean pumps in population of nicotine addicts. The partial correlation data showed consistent result (see the partial correlation analysis in supplemental materials). These results suggest that changes in the RSFC of the salience network (i.e., the dACC-thalamus) may be the potential underlying neural mechanism of risky decision making and nicotine dependence in population of nicotine addicts. The thalamus carries feedback from the basal ganglia to the dACC^37^. The dACC, which is part of the salience network and motor planning circuits^36^ and is associated with increased risk-seeking decisions^38^, initiates appropriate control signals to regulate behavior^39^. Combined with our results, these findings suggest that severely dependent smokers might engage in enhanced approach motivation when seeking motivationally salient stimuli, leading to an increase in risky behavior.

Although we also tested other RSFCs, we did not find any couplings that significantly correlated with both the mean pumps and the FTND score (see details in the supplemental materials). Two dissociable networks are involved in a large-scale brain network called the task-positive network^40^: one, termed the salience network, is anchored in the dACC and anterior insula, whereas the second is composed of the parietal and lateral prefrontal regions and is termed the executive control network^39^. In this study, we found RSFCs that were significantly correlated with both the mean pumps and the FTND score only between key nodes of the salience network (i.e., RSFCs of dACC-thalamus). We did not observe these results within the executive control network, suggesting that a specific circuit-level change in the salience network might be the underlying neural mechanism of risky decision making and nicotine dependence in population of nicotine addicts.

In addition, the RSFC of the dACC-thalamus positively correlated with severity of nicotine dependence. According to cognitive control deficit theory^41^, poor self-control ability of drug addicts may result in drug use. It is therefore expected that the activation of regions of the frontal cortex such as the DLPFC^4^, which plays a key role in cognitive control^42^, should be inversely correlated with severity of nicotine dependence. According to the incentive sensitization theory^43^ and the interoception theory^44^, severely dependent smokers show increased activation of the insula and dACC as compared to less-dependent smokers, which may represent increased interoceptive processing and motor preparation that lead to motivational drive to smoke^44^,^45^. In the present study, we found positive correlation between the RSFC of the dACC-thalamus and severity of nicotine dependence, which may support the incentive sensitization theory and the interoception theory. However, we did not find significant task-based activation of the frontal cortex^4^,^42^. But, the frontal cortex had a trend to activate (see the details in supplementary materials), making it difficult to explain the cognitive control deficit theory, and more evidence is needed to support this theory.

In previous studies, smokers took more pumps than non-smokers on the BART^1^,^46^. Thus, we inferred that smokers were more likely to take risks than non-smokers. In the population of nicotine addicts, we found a positive correlation between the FTND and the mean pumps, suggesting that severity of nicotine dependence may influence risk taking.

This study had several limitations. Firstly, the lack of a control group of non-smokers made it hard to directly assess whether smokers actually took more risks and had stronger connectivity of salience network than non-smokers. Our results showed that severity of nicotine dependence could influence risk taking behavior by influencing connectivity of salience network in population of nicotine addicts. Our results suggest that circuit-level changes in the salience network might be the underlying neural mechanism of nicotine addiction and abnormal risky decision making. However, more studies are required to conclusively ascertain this claim. Secondly, some decision-making phases, such as anticipation and selection, cannot be isolated by BART. However, the present study focused on the influence of risk level on risky decision making rather than on specific decision-making phases. Thirdly, only one female participated in the present study, so we could not analyze the role of gender. Though gender was not considered in this study, it might be an important factor and merits further research in the future. Fourthly, the difficulty of decision under inflation, which increased with increasing risk levels, could not be isolated. Difficulty is an important factor that should be considered before making a decision. In addition, anticipated rewards and losses are also considered in evaluation of risk-taking. While risk-taking is a result of evaluation of many variables, this specific variable (e.g., difficulty) was not considered in this study.

In the present study, we found that circuit-level connectivity between key nodes of the salience network, which impacts risky decision making, is associated with severity of nicotine dependence. These results suggest that in population of nicotine addicts, circuit-level changes in the salience network might be the underlying neural mechanism of nicotine dependence and risky decision making. Thus, interventions, such as transcranial direct current stimulation and transcranial magnetic stimulation, to balance the functional connectivity of the salience network may enhance the treatments for risky decision making and nicotine addiction.

## Materials and Methods

### Participants

Internet advertisements were used to recruit healthy daily smokers (>10 cigarettes per day for at least 1.5 years). Twenty-one smokers (mean age: 26.38 ± 4.31, range 18–40 years, 1 female) participated in the study. All participants had normal or corrected-to-normal vision and were right-handed. Potential participants were excluded if they reported a diagnosis of a psychiatric disorder, the use of addictive drugs (except nicotine), a prior head injury, or any contraindications for participating in an MRI study (e.g., non-removable metallic implants). The severity of nicotine dependence was assessed using the Fagerström Test of Nicotine Dependence [FTND^47^]. The study was approved by the Research Ethics Committee of the University of Science and Technology of China, and written informed consent in agreement with the Declaration of Helsinki was obtained from all participants. The methods and procedures were carried out in accordance with the approved guidelines.

### Task and procedure

The implementation of BART was modified from a previous study^4^. During this task, participants pressed buttons to sequentially pump a computer-simulated balloon image. Pressing the right button led to two possible outcomes that were immediately fed back to the participants: a no-explosion balloon pumping event in which a larger balloon with an increased wager was displayed, or a loss event in which a picture of an exploding balloon and the text “You Lose!” was displayed immediately. If the balloon exploded, participants would lose the wager and the virtual monetary amount lost was subtracted from the cumulative earnings as a penalty. If the participants chose to discontinue pumping (pressing the left button), a cash-out event in which the text “You Win!” was displayed and the amount of the reward was added to the cumulative earnings.

There was a jittered 2–4 s interval prior to the beginning of each balloon and a jittered 1.5–2.5 s interval between two continuous pumps. Twelve pump responses were possible for a given balloon, with a parametric increase in the probability of explosion over successive pump responses (i.e., take a risk with 0% probability of explosion for ¥0.0; 2.1% for ¥0.05; 4.2% for ¥0.15; 6.3% for ¥0.25; 14.6% for ¥0.55; 23.9% for ¥0.95; 31.3% for ¥1.45; 43.8% for ¥2.05; 56.3% for ¥2.75; 68.8% for ¥3.45; 79.2% for ¥4.25; and 89.6% for ¥5.15). The maximum number of pumps and the exact probability of explosion associated with a given inflation were not provided to participants. The risk level in the BART was directly and ecologically defined as the probability of explosion for each balloon.

### Behavior analysis

Consistent with previous studies^2^,^4^,^31^, risky decision making in this task is typically measured as the mean number of pumps, or “mean pumps”, across trials for balloons that did not explode. To investigate whether the nicotine addicts’ risky decision making was specifically affected by the severity of nicotine dependence, we used Pearson correlation analysis to compute the correlation between the FTND score and the mean pumps.

### MRI data acquisition

Gradient echo-planar magnetic resonance imaging data were obtained during the whole task procedure on a 3T Siemens Magnetom Trio scanner (Siemens Medical Solutions, Erlangen, Germany) at the Anhui Provincial Hospital. Task stimuli were projected onto a display screen at the back of the magnet’s bore, and participants viewed the stimuli through a mirror. Button-press responses on an fMRI-compatible response box were made using the left and the right thumbs. A circularly polarized head coil was used for radio frequency transmission and reception, with foam padding to restrict head motion.

Resting-state fMRI data (240 volumes, 8 minutes) were acquired by asking participants to keep their eyes closed and to remain at rest. This was followed by another 8 minute functional scan corresponding to BART. Functional images were acquired with a T2*-weighted echo-planar imaging sequence (TE = 30 ms, TR = 2000 ms, FOV = 240 mm, Matrix = 64 × 64, flip angle = 85°) with 33 axial slices (no gaps, voxel size: 3.75 × 3.75 × 3.7 mm^3^) covering the whole brain. In addition, high-resolution T1-weighted spin-echo images were also acquired.

### Image data preprocessing

The imaging data were processed using AFNI [Analysis of Functional Neuroimages^48^] and MATLAB (version 7.6.0.324; Mathworks). Each participant’s raw data were corrected for temporal shifts between slices, corrected for motion, spatially smoothed with a Gaussian kernel (full width at half maximum = 8 mm), and temporally normalized (for each voxel, the signal of each volume was divided by the temporally averaged signal).

### Analysis of functional imaging data

Three types of events were included in the general linear model of the BART task: pumps, cash outs and balloon explosions. Each type of event included two regressors in the general linear model. For the pumps, which represented risk-taking in this task, two regressors were defined: (1) Pumps_parametric_, the only regressor we were interested in, which represented the parametrically modulated activity by the risk level associated with each pump (i.e., the probability of explosion) and modeled all of the pump events at the onset time of pumping; and (2) Pumps_avg_, the averaged activity across all pumps without parametric modulation by the individual average risk level (i.e., the mean pumps correspond to the probability of explosion) using the same onset time^2^. For a cash-out event, two regressors (similar to pumps) were defined: (1) Cash-out_parametric_; and (2) Cash-out_avg_. For an explosion event, two regressors similar to pumps were defined: (1) Explosion_parametric_; and (2) Explosion_avg_.

These six regressors were convolved with the gamma function to approximate the hemodynamic response of the brain. Head motion parameters in 6 directions were also included as covariates. The resultant z-statistic map (transferred from the t-value map from the GLM result) of Pumps_parametric_ (i.e., the risk level) was transformed to the Talairach space. To test the activation of risk level, a group-level one-sample *t*-test was used and the responses were identified if they survived whole brain correction for family-wise errors at a cluster-level threshold of p < 0.005 (cluster size 30 voxels, 810 mm^3^) and a voxel-level threshold of p < 0.0001.

### Risk level-related activation and relationship to task performance and severity of nicotine dependence

For the z-statistic map of risk level, the averaged z-value of every voxel within each significantly activated region related to risk level was calculated for each participant. Then, Pearson correlation analysis was used to assess the relationship between risk level-related brain activation and task performance as well as the severity of nicotine dependence.

The same analysis was also applied to cash-out and explosion events to clarify to influence of outcome on risky decision making and the results were provided in the supplementary materials.

### RSFC and its relationship to task performance and severity of nicotine dependence

For the resting-state fMRI data, we regressed the motion data out of the time series and performed bandpass temporal filtering (0.01–0.0 Hz) on the residual signals. Then, to further reduce nuisance signals, the average white matter and CSF signals were regressed out. The white matter mask was determined from the high-resolution structural image using the FAST segmentation program (Functional MRI of the Brain software library; www.fmrib.ox.ac.uk). Each participant’s CSF mask was manually drawn to fit the anatomical boundaries of a standardized Talairach atlas brain, which was transformed onto the image space of each participant and modified according to the cortical structures of the participant’s brain by referencing the anatomical boundaries in the high-resolution structural image. These nuisance signals were used to account for fluctuations that were unlikely to be relevant to neuronal activity^49^. Then, data scrubbing was performed, and any volume with a framewise-dependent value for the temporal derivative of a time course’s root mean squared head motion variance exceeding 0.5 was excluded. The resultant resting-state fMRI data were then used for functional connectivity analysis.

We defined the AI, dACC, and thalamus activated by risk level during BART as regions of interest (ROIs) in the RSFC analysis. The preprocessed resting-state fMRI time series were averaged within each ROI. Correlations between the averaged ROI time series were calculated and then transformed to Fisher z-values. Finally, the correlations between RSFCs and the mean pumps and the severity of nicotine dependence were calculated. We performed a FDR-correction for multiple comparisons (FDR < 0.05).

### Mediation analysis

To examine whether the RSFC of the dACC-thalamus pathway might mediate the effect of the FTND score on mean pumps, we performed a causal mediation analysis^24^,^25^. First, the hypothesized mediator (the RSFC of the dACC-thalamus) was regressed on the independent variable (FTND score); second, the dependent variable (mean pumps) was regressed on the independent variable; and third, the dependent variable was regressed on both the independent variable and the hypothesized mediator in one equation. Coefficients for the equation were estimated, and mediation was considered to occur if the relationship between the independent and dependent variables could be partially or totally accounted for by the hypothesized mediator.

## Additional Information

**How to cite this article**: Wei, Z. *et al*. Resting-state functional connectivity between the dorsal anterior cingulate cortex and thalamus is associated with risky decision-making in nicotine addicts. *Sci. Rep.* **6**, 21778; doi: 10.1038/srep21778 (2016).

## Supplementary Material

Supplementary Information

## Figures and Tables

**Figure 1:**
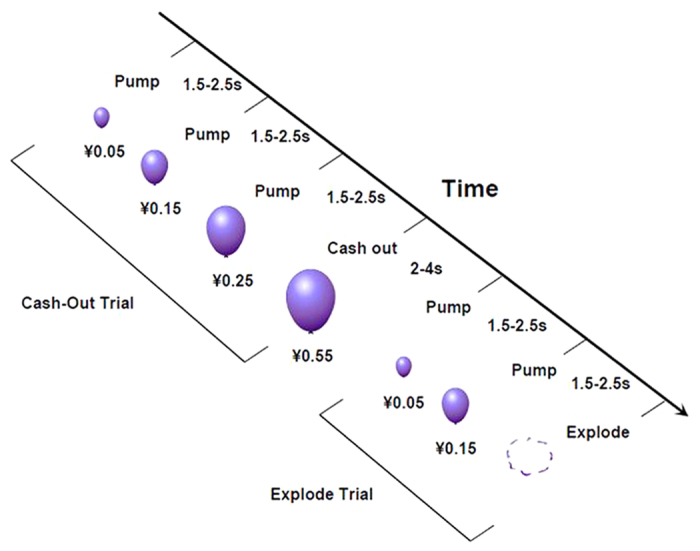
The BART paradigm.

**Table 1:** The risk level-related activation derived from a whole-brain analysis and its relationship to the mean pumps and severity of nicotine dependence.

Regions	x	y	z	Max z	Cluster size (voxels)	Mean pumps		FTND	
						r	p	r	p
R precentral gyrus	−34	14	52	5.35	249	0.288	0.205	−0.008	0.972
R anterior insula	−32	−23	−1	6.65	146	0.399	0.074	0.192	0.405
Bilateral dACC	−8	−2	47	7.20	132	0.368	0.101	0.180	0.435
R IPL	−56	25	32	4.59	82	0.244	0.286	0.034	0.883
Bilateral thalamus	−8	22	−4	3.60	63	0.305	0.179	0.152	0.511
L anterior insula	31	−17	11	6.07	42	0.335	0.138	0.260	0.255
L IPL	55	28	35	4.47	40	0.257	0.262	0.011	0.961
