# Supplementary material for: Resting-state functional connectivity between the dorsal anterior cingulate cortex and thalamus is associated with risky decision-making in nicotine addicts
Source: Sci Rep. 2016 Feb 16;6:21778. doi: 10.1038/srep21778 (PMC4755012; doi:10.1038/srep21778)
Supplement: Supplementary Information [file srep21778-s1.doc]

**Resting-state functional connectivity between the dorsal anterior cingulate cortex and thalamus is associated with risky decision-making in nicotine addicts**

Zhengde Wei1, +, Nannan Yang1, +, Ying Liu2, *, Lizhuang Yang1, Ying Wang1, Long Han1, Rujing Zha1, Ruiqi Huang1, Peng Zhang3, Yifeng Zhou1, Xiaochu Zhang1, 4, 5, 6, *

1 Key Laboratory of Brain Function and Disease, Chinese Academy of Sciences, School of Life Sciences, University of Science & Technology of China, Hefei, Anhui 230027, China

2 Provincial Hospital Affiliated to Anhui Medical University, Hefei, Anhui 230001, China

3 State Key Laboratory of Brain and Cognitive Science, Institute of Biophysics, Chinese Academy of Sciences, Beijing, 100101, China

4 School of Humanities & Social Science, University of Science & Technology of China, Hefei, Anhui 230026, China

5 Center of Medical Physics and Technology, Hefei Institutes of Physical Science, CAS, Hefei, Anhui 230031, China

6 Centers for Biomedical Engineering, University of Science & Technology of China, Hefei, Anhui 230027, China

+ These authors contributed equally to this work

 Correspondence: [zxcustc@ustc.edu.cn](mailto:zxcustc@ustc.edu.cn); felice828@126.com

**Contents:**

**I. Brain activations of Cash-outparametric and Explosion­parametric**

**II. Partial correlation analysis**

**III. Other RSFCand relationship to mean pumps and FTND score**

**IV. Whole brain RSFC analysis using the striatum as seed and relationship to mean pumps and FTND score**

**V. Whole brain RSFC analysis using the dACC, thalamus and insula as seeds and relationship to mean pumps and FTND score**

**VI. Risk level-related activation in DLPFC**

**I. Brain activations of Cash-outparametric and Explosion­parametric**

To test activations of Cash-outparametric, a group-level one-sample *t*-test was used and responses were identified if they survived whole brain correction for family-wise error at a cluster-level threshold of p < 0.05 (cluster size 45 voxels, 1215 mm3) and a voxel-level threshold of p < 0.001 (no significant activations of Cash-outparametric were found at a cluster-level threshold of p < 0.005). Significant activations were found in the right declive/fusiform gyrus, the left declive/lingual gyrus, the right anterior insula, and the right posterior cingulate cortex (**Fig s1**). None of these brain activations were associated with mean pumps or the severity of nicotine dependence (**Table s1**). While the right anterior insula was a key node of the salience network, we also tested the relationship between activation of Cash-outparametric in the right anterior insula and RSFC among the rAI-dACC/thalamus/lAI network. However, activation of Cash-outparametric in the right anterior insula did not correlate with RSFC of rAI-dACC/thalamus/lAI (r = 0.230, p = 0.316; r = - 0.073, p = 0.754; r = - 0.160, p = 0.488). These results suggest that the salience network may modulate the processing of risk level, but not the outcome of the cash-out.

No significant activation of Explosion­parametric (p < 0.005, family-wise error corrected) was found. One reason could be that the event frequency of explosion was too low (7.2±2.7) to have enough statistical power.


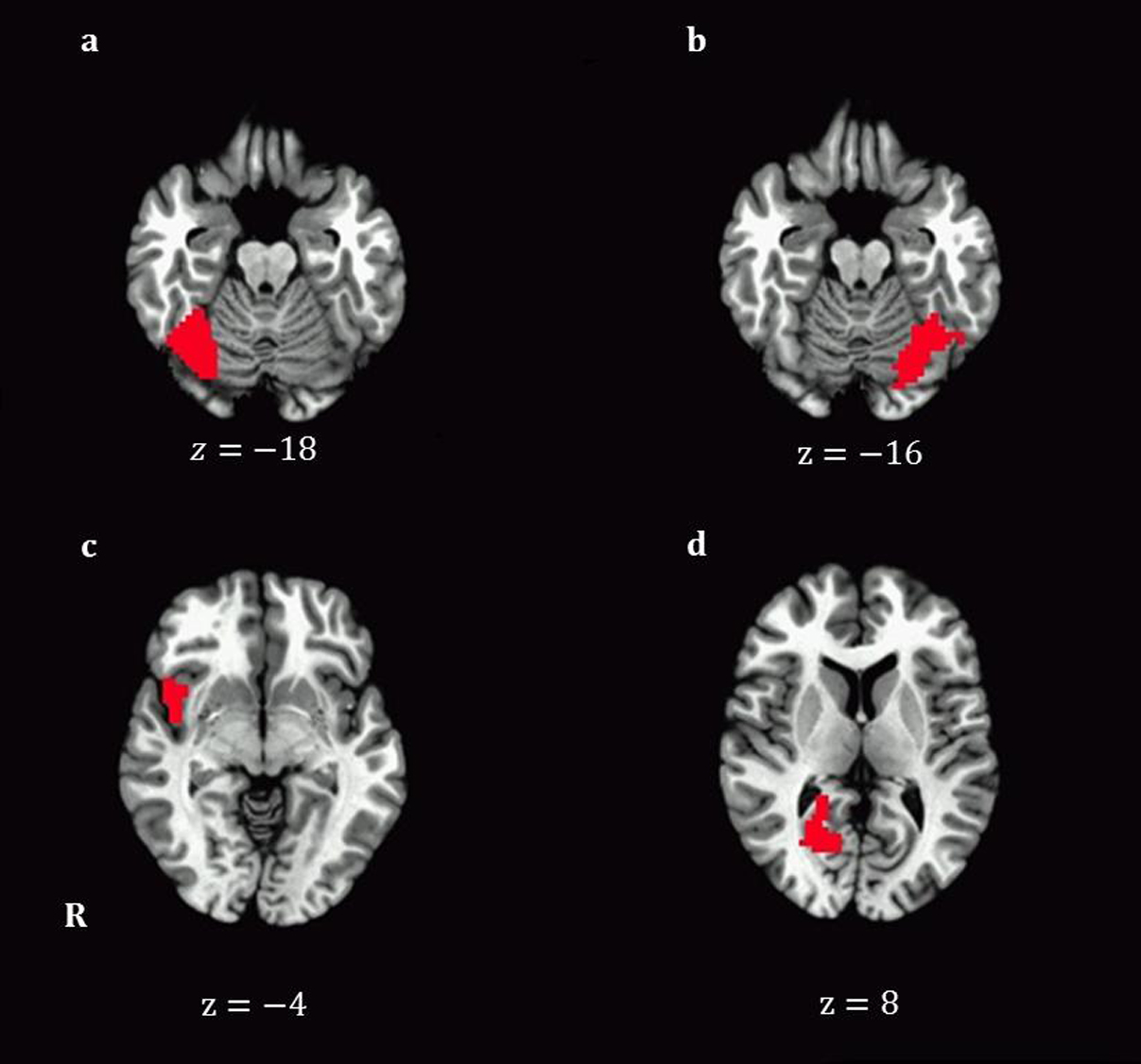


**Figure s1** Brain activation by Cash-outparametric during risky decision making. During risky decision making, brain activation by Cash-outparametric was found in **a** right declive/fusiform gyrus, **b** left declive/lingual gyrus, **c** right anterior insula and **d** right posterior cingulate cortex.

**Table s1** Relationship between activations of Cash-outparametric and mean pumps / nicotine dependence severity

|  | Mean pumps | |  | FTND | |
| --- | --- | --- | --- | --- | --- |
| Regions | r | p |  | r | p |
| R declive/fusiform gyrus | 0.223 | 0.332 |  | -0.108 | 0.640 |
| L declive/lingual gyrus | 0.089 | 0.701 |  | -0.203 | 0.379 |
| R anterior insula | 0.195 | 0.397 |  | -0.137 | 0.553 |
| R posterior cingulate cortex | - 0.135 | 0.558 |  | -0.158 | 0.495 |

**II. Partial correlation analysis**

To support the opinion that the RSFC of the dACC-thalamus might underlie the neural mechanism of abnormal risky decision making in nicotine addicts, a partial correlation analysis was performed. The FTND score did not correlate with the mean pumps while controlling for the influence of RSFC in the dACC-thalamus pathway [r (18) = 0.111, p = 0.642]. This suggests that the RSFC of the dACC-thalamus pathway may be the link between risky decision making and nicotine addiction. This result was consistent with the mediation effect observed in the present study, in which dACC-thalamus coupling fully mediated the effect of severity of nicotine dependence on mean pumps.

**III. Other RSFCand relationship to mean pumps and FTND score**

RSFC between regions related to risk level except couplings between bilateral dACC, r-AI, l-AI and bilateral thalamus were calculated and then relationship to mean pumps and FTND score was tested. The results are shown in **Table s2**. We did not find any coupling which significantly correlated with both mean pumps and FTND score.

**Table s2** Relationship between other RSFC and mean pumps / nicotine dependence severity

|  | Mean pumps | |  | FTND | |
| --- | --- | --- | --- | --- | --- |
| Regions | r | p |  | r | p |
| rPG-dACC | 0.397 | 0.075 |  | 0.237 | 0.301 |
| rIPL-dACC | 0.428 | 0.053 |  | 0.185 | 0.422 |
| lIPL-dACC | 0.339 | 0.133 |  | -0.194 | 0.401 |
| rAI-rPG | 0.420 | 0.058 |  | 0.275 | 0.228 |
| rIPL-rPG | 0.349 | 0.121 |  | 0.303 | 0.182 |
| thalamus-rPG­­ b | 0.420 | 0.058 |  | 0.529 | 0.014 |
| lAI-rPG a | 0.438 | 0.047 |  | 0.215 | 0.350 |
| lIPL-rPG | 0.251 | 0.273 |  | 0.255 | 0.265 |
| rIPL-rAI a | 0.480 | 0.028 |  | 0.311 | 0.171 |
| lIPL-rAI a | 0.517 | 0.016 |  | -0.097 | 0.675 |
| thalamus-rIPL | 0.304 | 0.181 |  | 0.331 | 0.143 |
| lAI-rIPL a | 0.631 | 0.002 |  | 0.214 | 0.351 |
| lIPL-rIPL | 0.054 | 0.818 |  | -0.115 | 0.620 |
| lIPL-thalamus | 0.346 | 0.124 |  | 0.331 | 0.142 |
| lIPL-lAI a | 0.506 | 0.019 |  | 0.083 | 0.720 |

a significant correlation with mean pumps; b significant correlation with FTND score

**IV. Whole brain RSFC analysis using the striatum as seed and relationship to mean pumps and FTND score**

Striatum, a key region of reward system, is also critical for risky decision making and the dopamine regulation in striatum is an important mechanism underlying this process[1-3](#_ENREF_1). Greater risk taking has been associated with higher dopamine D1 receptor mRNA expression in striatum in rats[4](#_ENREF_4). Recent studies have shown that the reward system, especially striatal function, is altered in drug addiction, which may mediate increase in risky decision making[5-7](#_ENREF_5). In the present study, we also tested whether connectivity of reward system centered on striatum influenced risky decision making in population of nicotine addicts.

In order to obtain risk level-related activation in striatum, we lowered the statistical threshold (p < 0.01, family-wise error corrected) and the bilateral striatum survived (Talairach [x, y, z], left [10, -2, 2]; right [-14, -2, 5]). We performed a whole brain analysis using the striatum as seed and then assessed its correlation with the risk taking behavior and the FTND score. The results are shown in **table s3** (p < 0.005, uncorrected, cluster size 15 voxels, 405 mm3). No coupling significantly correlated with both the FTND score and the mean pumps.

Striatum is critical for risky decision making and the dopamine regulation in the striatum is an important mechanism underlying this process[1-3](#_ENREF_1). We found that couplings of striatum RSFC correlated with risk taking behavior, which supported the importance of striatum in risky decision making. We also found that couplings of striatum RSFC correlated with the severity of nicotine dependence. A number of studies have shown that striatal function is altered in drug addiction[5-7](#_ENREF_5). However, no couplings of striatum RSFC correlated with both severity of nicotine dependence and risk taking behavior, which may be ascribed to the differences between nicotine trait- and state-dependent effects on reward processing. For example, nicotine can induce both acute and chronic hypersensitivity of the DA reward system[8](#_ENREF_8). Nicotine alters the decision making involved the reward process, which seems to be the effect of state change (e.g., craving and acute influence of nicotine exposure)[8-10](#_ENREF_8), while the FTND is smoking-related stable and heritable trait[11](#_ENREF_11). It is probable that nicotine trait- and state-dependent effects may influence the reward system but on separate parts, which may be a possible reason why no couplings of striatum RSFC linked between risk taking behavior and severity of nicotine dependence.

**V. Whole brain RSFC analysis using the dACC, thalamus and insula as seeds and relationship to mean pumps and FTND score**

We performed a whole brain analysis using the dACC, thalamus and insula as seeds and then assessed the correlation with risk taking behavior and the FTND score. The results are shown in **table s 3** (p < 0.005, uncorrected, cluster size 15 voxels, 405 mm3). We did not find any couplings outside the salience network that correlated with both the FTND score and the mean pumps.

**Table s3** Relationship between whole brain analysis of RSFC and mean pumps / nicotine dependence severity

|  |  |  |  |  | Cluster size (voxels) |
| --- | --- | --- | --- | --- | --- |
| Regions | x | y | z | Max z |
| **Dorsal ACC** |  |  |  |  |  |
| **Correlation with FTND** |  |  |  |  |  |
| R insula/inferior frontal gyrus | -53 | -11 | 8 | 4.08 | 45 |
| Bilateral thalamus | 1 | 22 | -1 | 4.03 | 35 |
| L parahippocampal gyrus | 16 | 37 | 5 | -3.58 | 35 |
| Bilateral rostral ACC | 1 | -11 | -7 | -3.60 | 17 |
| R caudate/cingulate cortex | -8 | 10 | 26 | 3.48 | 16 |
| **Correlation with mean pumps** |  |  |  |  |  |
| Bilateral paracentral lobule | -8 | 37 | 71 | -4.60 | 173 |
| L fusiform gyrus | 34 | 55 | -4 | 3.91 | 96 |
| R insula/inferior frontal gyrus | -44 | -11 | 17 | 3.69 | 75 |
| L inferior parietal lobule | 31 | 40 | 41 | 3.84 | 62 |
| Bilateral medial frontal gyrus | 1 | -62 | 5 | -3.59 | 37 |
| L parahippocampal gyrus | 19 | 16 | -10 | -3.74 | 24 |
| R middle temporal gyrus | -41 | 70 | 11 | 3.09 | 18 |
| **R insula** |  |  |  |  |  |
| **Correlation with FTND** |  |  |  |  |  |
| Bilateral posterior cingulate | 4 | 49 | 11 | -3.95 | 33 |
| R middle frontal gyrus | -41 | 1 | 53 | 4.08 | 31 |
| R precuneus | -17 | 67 | 29 | 3.85 | 28 |
| L angular gyrus | 31 | 55 | 35 | 3.30 | 19 |
| L superior frontal gyrus | 7 | -56 | 26 | -3.19 | 17 |
| **Correlation with mean pumps** |  |  |  |  |  |
| L inferior parietal lobule | 43 | 34 | 35 | 4.20 | 211 |
| L precentral gyrus | 28 | 16 | 53 | 3.74 | 120 |
| R inferior parietal lobule | -44 | 34 | 44 | 3.98 | 100 |
| R precentral gyrus | -41 | 7 | 41 | 3.62 | 92 |
| L precuneus | 19 | 58 | 35 | 4.06 | 36 |
| L middle frontal gyrus | 46 | -2 | 44 | 3.58 | 33 |
| R caudate | -14 | 1 | 17 | 3.54 | 27 |
| **R thalamus** |  |  |  |  |  |
| **Correlation with FTND** |  |  |  |  |  |
| R dACC | -5 | 4 | 47 | 3.22 | 16 |
| **Correlation with mean pumps** |  |  |  |  |  |
| Bilateral dACC | 7 | -2 | 41 | 3.51 | 16 |
| **L insula** |  |  |  |  |  |
| **Correlation with FTND** |  |  |  |  |  |
| L angular gyrus | 31 | 55 | 35 | 3.73 | 46 |
| L medial frontal gyrus | 10 | -56 | 11 | -3.56 | 38 |
| R middle frontal gyrus | -50 | -23 | 35 | 3.52 | 19 |
| Correlation with mean pumps |  |  |  |  |  |
| L insula/inferior frontal gyrus | -56 | -8 | 17 | 5.26 | 176 |
| L inferior parietal lobule | 43 | 34 | 35 | 3.86 | 164 |
| R inferior parietal lobule | -35 | 49 | 44 | 3.83 | 61 |
| R postcentral gyrus | -53 | 28 | 38 | 3.80 | 40 |
| R superior temporal gyrus | -62 | 28 | 11 | 3.90 | 35 |
| L precentral gyrus | 49 | 1 | 35 | 3.89 | 30 |
| L precuneus | 19 | 55 | 35 | 3.85 | 18 |
| **R striatum** |  |  |  |  |  |
| **Correlation with FTND** |  |  |  |  |  |
| L inferior temporal gyrus | 58 | 13 | -22 | 3.96 | 22 |
| Bilateral thalamus | -2 | 7 | -4 | 3.75 | 22 |
| R superior temporal gyrus | -23 | -14 | -25 | 3.58 | 15 |
| **Correlation with mean pumps** |  |  |  |  |  |
| R ACC | -23 | -11 | 23 | -3.32 | 20 |
| **L striatum** |  |  |  |  |  |
| **Correlation with FTND** |  |  |  |  |  |
| L declive | 19 | 79 | -22 | -3.12 | 25 |
| **Correlation with mean pumps** |  |  |  |  |  |
| L superior frontal gyrus | 19 | -17 | 50 | -3.92 | 130 |
| L middle frontal gyrus | 34 | -41 | 5 | -3.38 | 52 |
| L parahippocampal gyrus | 19 | 16 | -13 | -4.38 | 49 |
| R parahippocampal gyrus | -29 | 13 | -16 | -3.45 | 36 |
| R occipital gyrus | -29 | 88 | -10 | 3.31 | 25 |
| R superior frontal gyrus | -2 | -53 | 32 | -3.66 | 25 |
| R precentral gyrus | -41 | 7 | 35 | 3.16 | 19 |
| R occipital gyrus | -44 | 70 | -7 | 2.99 | 15 |

**VI. Risk level-related activation in DLPFC**

Based on previous study (Rao et al., NeuroImage, 2008), the bilateral DLPFC ROIs were comprised of two 5mm spheres MNI coordinates 30/-34, 36/46, 20/16. A one-sample *t*-test was used to test the activation of risk level in these two ROIs. Our results showed that risk level-related activation was found in bilateral DLPFC (rDLPFC: p = 0.0013; lDLPFC: p = 0.0331), suggesting that risk level-related activation in smokers may be similar with normal controls. Meanwhile, activation in DLPFC did not correlate with the FTND score or the mean pumps.

References

1. Ernst, M. et al. Choice selection and reward anticipation: an fMRI study. *Neuropsychologia* **42**, 1585-1597 (2004).

2. Matthews, S.C., Simmons, A.N., Lane, S.D. & Paulus, M.P. Selective activation of the nucleus accumbens during risk-taking decision making. *Neuroreport* **15**, 2123-2127 (2004).

3. Tom, S.M., Fox, C.R., Trepel, C. & Poldrack, R.A. The neural basis of loss aversion in decision-making under risk. *Science* **315**, 515-518 (2007).

4. Simon, N.W. et al. Dopaminergic modulation of risky decision-making. *J Neurosci* **31**, 17460-17470 (2011).

5. Nestor, L., Hester, R. & Garavan, H. Increased ventral striatal BOLD activity during non-drug reward anticipation in cannabis users. *NeuroImage* **49**, 1133-1143 (2010).

6. Hyatt, C.J. et al. Reward-related dorsal striatal activity differences between former and current cocaine dependent individuals during an interactive competitive game. *PLoS One* **7**, e34917 (2012).

7. Yamamoto, D.J. et al. Temporal profile of fronto-striatal-limbic activity during implicit decisions in drug dependence. *Drug Alcohol Depend* **136**, 108-114 (2014).

8. Kenny, P.J. & Markou, A. Nicotine self-administration acutely activates brain reward systems and induces a long-lasting increase in reward sensitivity. *Neuropsychopharmacology* **31**, 1203-1211 (2006).

9. Janes, A.C., Farmer, S., Peechatka, A.L., Frederick Bde, B. & Lukas, S.E. Insula-Dorsal Anterior Cingulate Cortex Coupling is Associated with Enhanced Brain Reactivity to Smoking Cues. *Neuropsychopharmacology* **40**, 1561-1568 (2015).

10. Phillips, P.E., Stuber, G.D., Heien, M.L., Wightman, R.M. & Carelli, R.M. Subsecond dopamine release promotes cocaine seeking. *Nature* **422**, 614-618 (2003).

11. Li, M.D. Identifying susceptibility loci for nicotine dependence: 2008 update based on recent genome-wide linkage analyses. *Human Genetics* **123**, 119-131 (2008).
